# WebScipio: An online tool for the determination of gene structures using protein sequences

**DOI:** 10.1186/1471-2164-9-422

**Published:** 2008-09-18

**Authors:** Florian Odronitz, Holger Pillmann, Oliver Keller, Stephan Waack, Martin Kollmar

**Affiliations:** 1 Max-Planck-Institut für Biophysikalische Chemie, Abteilung NMR-basierte Strukturbiologie, Am Fassberg 11, 37077 Göttingen, Germany; 2 Universität Göttingen, Institut für Informatik, Lotzestr. 16-18, 37083 Göttingen, Germany

## Abstract

**Background:**

Obtaining the gene structure for a given protein encoding gene is an important step in many analyses. A software suited for this task should be readily accessible, accurate, easy to handle and should provide the user with a coherent representation of the most probable gene structure. It should be rigorous enough to optimise features on the level of single bases and at the same time flexible enough to allow for cross-species searches.

**Results:**

WebScipio, a web interface to the Scipio software, allows a user to obtain the corresponding coding sequence structure of a here given a query protein sequence that belongs to an already assembled eukaryotic genome. The resulting gene structure is presented in various human readable formats like a schematic representation, and a detailed alignment of the query and the target sequence highlighting any discrepancies. WebScipio can also be used to identify and characterise the gene structures of homologs in related organisms. In addition, it offers a web service for integration with other programs.

**Conclusion:**

WebScipio is a tool that allows users to get a high-quality gene structure prediction from a protein query. It offers more than 250 eukaryotic genomes that can be searched and produces predictions that are close to what can be achieved by manual annotation, for in-species and cross-species searches alike. WebScipio is freely accessible at .

## Background

Gene prediction is one of the most important steps in analyzing genome sequences. Mostly, *de novo *gene prediction is based on sophisticated algorithms that model open reading frames, consensus splice sites, and start and stop codon sequences. Often additional data like EST (Expressed Sequence Tag) sequences or information from cross-species multiple sequence alignments are used. Cross-species DNA sequence comparisons are increasingly being used to identify both coding regions and functional DNA elements [1,2]. These functional elements might be promoter sequences, transcription factor binding sites, termination signals or other regulatory elements. Comparisons of sequences of multiple species have either been performed at a genomic level (e.g. [3,4]) or at the single gene and gene family scale (e.g. [5]).

One important aim of most of the large-scale comparative studies has been to improve the annotation of the genomes, like the identification of new genes [6] or new constitutive and alternative exons [7]. These studies have also resulted in the prediction of regulatory regions [8]. However, only a limited number of the conserved non-coding sequences that have been identified by these studies have been functionally characterized.

Cross-species DNA sequence alignments of entire genomes are available for several eukaryotes [9,10]. These genomes, however, cover only a small part of the about 330 eukaryotic genomes for which genome assemblies are available , as of Feb. 2008 [11]). Thus, a comparison of the genomic DNA sequences of a specific gene or gene family of a certain set of species would require a lot of time consuming manual steps. These involve obtaining the desired eukaryotic genome assemblies, identifying all homologous genes, and predicting their gene structures.

To retrieve gene data and non-coding sequence, some programs and web-tools are available. The Retrieval of Regulative Regions (RRE) tool is a Java application which parses annotation and homology data from NCBI [12]. RRE is available as a web application, which however only hosts a small number of eukaryotic genomes and annotation data only from NCBI, or requires local installation and local copies of the desired genome and annotation files. In addition, the non-coding sequences retrieval system (NCSRS) has been developed [13] which offers access to 16 genomes and annotation data from both Ensembl [14] and NCBI. Access to most vertebrates and some other eukaryotes is offered by Ensembl and the UCSC browser [15]. Both web interfaces allow to search for genes and to recover any part of the gene of interest. However, when searching the genomes with descriptive terms or accession numbers, the output is mainly based on results from the various gene prediction programs, although it is often supported by evidence from cDNA or manual curation. When using BLAST [16] or BLAT [17], the quality of the resulting gene structure is limited by the parameters of these programs. Of course there are further species-specific genome pages providing access to gene data. But there is no service offering the retrieval of the gene structures corresponding to protein queries of almost all sequenced and assembled eukaryotic genomes.

We have developed WebScipio, a web interface to the Scipio software [18], which can determine the precise gene structure given a protein sequence. WebScipio provides access to a continuously updated list of almost all eukaryotic genome assemblies that are available worldwide (for a comprehensive list see . Additionally, the user can retrieve all relevant data in human readable format in a very convenient way. For the integration with other programs, WebScipio provides a web service.

## Implementation

The web application is implemented in the Ruby programming language [19] using the Ruby on Rails [20] framework. The list of the genome files available for search are stored in an index file which is generated from the diArk database [11]. We are searching for new releases of genome data on a weekly basis, and as soon as new data is released it will be available to WebScipio. The index file contains metadata about the genomes, the sequencing projects and the species (see also species-selection section). As the user types into the text box, the index file is searched at every keystroke and the matching species are shown in a pulldown menu. The genome files are updated manually by regularly searching the various genome sequencing project pages for updates and new genomes. After a genome file is selected, one or several protein sequences are provided by the user, and the parameters for BLAT and Scipio are collected. The information needed for the run is then complete.

In order to improve the response time a script splits the genome file into several files and starts a predefined number of parallel BLAT jobs. This allows using the multi-core architecture of modern servers without re-implementing the BLAT algorithm. The resulting PSL-Files are concatenated and Scipio [18] is started to assemble and refine BLATs search results. Scipio saves the results as a YAML file [21]. YAML is a simple data serialization format which can store nested data structures. It is human readable and parsers exist for a great number of programming languages.

The YAML file is then parsed and a graphical representation is generated. WebScipio determines the optimal ratio for the scaling of exons and introns so that large intron sequences do not render the visualization useless. The pictures of the gene structure are generated as publication quality SVG (Scalable Vector Graphics [22]) but are automatically be converted to PNG (Portable Network Graphics [23]) if SVG is not supported by the browser.

The web service was also implemented using Ruby on Rails and can be used with RPC (Remote Procedure Call) or SOAP (Simple Object Access Protocol). The methods of the application programming interface resemble the functionality of the website. A normal usage would be to call a series of functions in order to arrive at a gene structure and a visualization: SearchSpecies, SearchGenomes, Query, GetSvg.

## Results and discussion

### Web interface

WebScipio offers a clean and simple web interface that can easily be used by inexperienced users. At the same time expert users have enough options to adjust the underlying algorithm to get the best possible results, even in difficult cases.

#### Species selection

**Figure 1:**
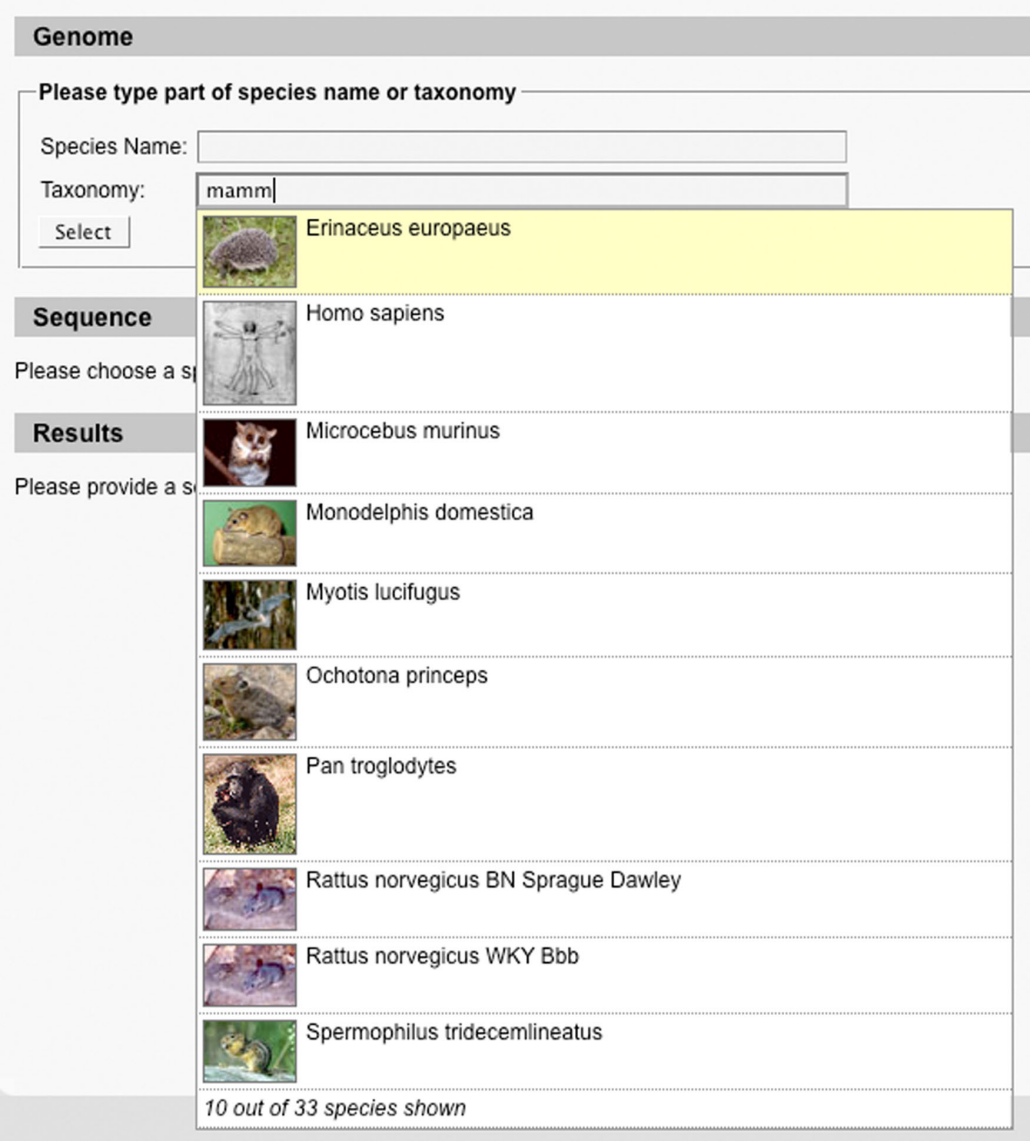
**Species selection**. The screenshot shows the species selection auto completion field. As the user types, species matching his query appear. Different types of information are taken into account when searching. In the example the user types 'mamm' and all Mammalia are listed.

Species are selected using an auto-completion field. The user starts typing and a selection of species matching the search term is shown. Apart from searching for the scientific name of a species, many different types of information can be searched for: Alternative scientific names, common names, anamorph names (for fungi), and taxonomy (Figure 1). Users can also search for abbreviations of sequencing centers (e.g. 'JGI' for Joint Genome Institute) or type of genome files (e.g. 'chromosome').

#### Genomes

WebScipio offers 751 genome files from 256 eukaryotes (as of Feb. 2008) for searching, which amounts to more than 360 gigabytes of sequence data. Genome data is kept up to date, but at the same time older versions are offered. Many different types of genome data can be searched: Chromosomes, supercontigs, contigs, unplaced reads/contigs as well as genome sequences from mitochondria, chloroplasts and apicoplasts, if available.

#### Protein query

The query for the search is one or several protein sequences, which are entered plain or in FASTA format.

#### Search options

**Figure 2:**
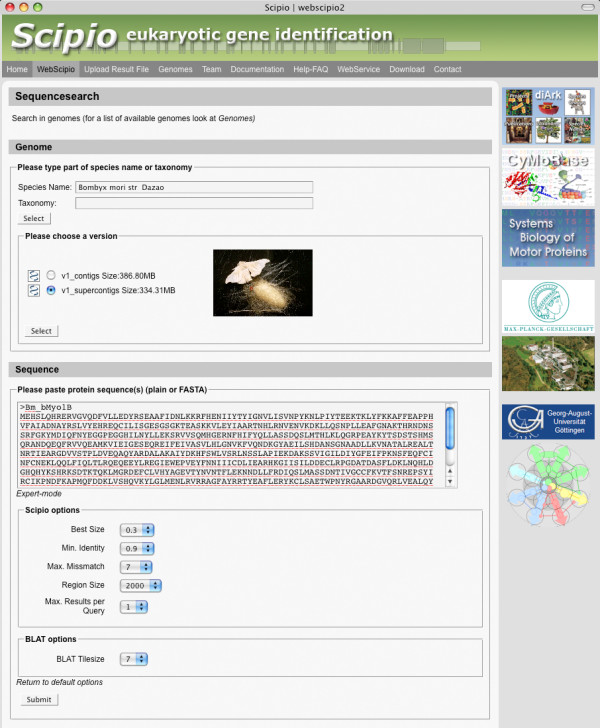
**Input interface**. The screenshot shows the input interface of WebScipio. First the user chooses a species, then a genome, enters the query sequence and then specifies optional search parameters.

The search options define how tolerant the algorithm is regarding contigs and exons (Figure 2). 'Best Size' defines the minimum fraction of the query that has to be found on one single contig. If, for example, the genome sequence is in an early stage of assembly and highly fragmented, the largest part that is found on one contig might only be 20% of the query. 'Min Identity' defines the minimal identity within a stretch of DNA in order to be taken into account by WebScipio. 'Max Mismatch' defines the maximum number of mismatches between the query sequence and a contig in order to be included in the results. The values for these parameters largely depend on the quality of the genome. 'Region Size' defines the length of the up-and downstream regions that can be retrieved. 'BLAT Tilesize' determines the width of the search window used to scan the genome. Decreasing this value makes it more likely that small exons are found but also slows down the search process.

#### Visualization

**Figure 3:**
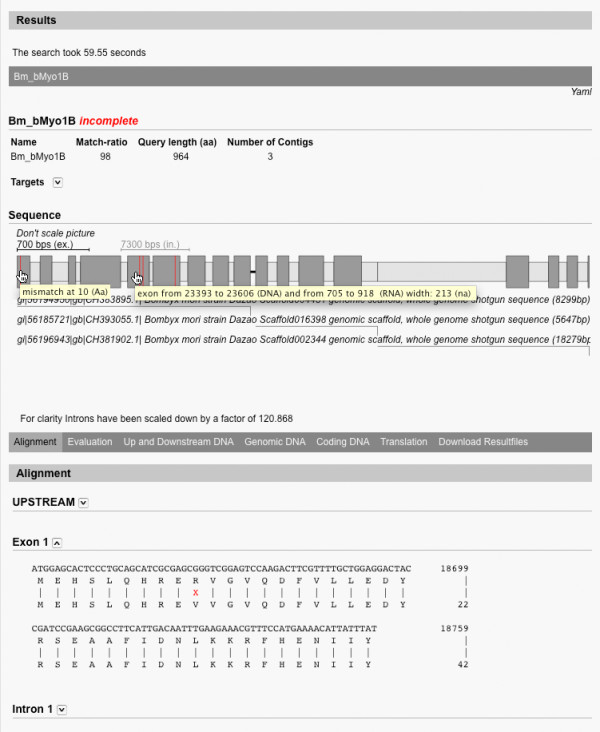
**Result view**. The screenshots shows the result view for a query. Basic statistics are provided along with a visualisation of the gene structure showing introns, exons, mismatches, frameshifts, and gaps. It also shows which part of the gene was found on which contig. Tooltips provide further detail. Below, the alignment view is shown, clearly highlighting sites of disagreement.

A characteristic of conventional spliced alignment tools is that they produce lists of hits, maybe alongside with basic graphics, but most of the time the user does not see at a glance, what the gene structure might be. WebScipio generates a graphical representation of the gene that clearly indicates the length and position of exons and introns and shows, where discrepancies are located. It also shows the identifiers of the target sequences (Figure 3). In order not to make small exons vanish when very large intronic stretches are found, the scaling of introns and exons is automatically balanced to make the picture visually meaningful. Tooltips show additional information.

#### Alignments, DNA and target translation

For detailed inspection of the hits, WebScipio generates an easy to read alignment of the query and the genome. It is grouped by exons, and mismatches and frame shifts are highlighted. Different stretches of DNA can be viewed: Up- and downstream DNA, genomic DNA from the first to the last exon including introns, or the coding DNA. The translation of the coding DNA as determined by the algorithm can also be viewed.

#### File download

Six types of files can be downloaded: A FASTA file containing all types of DNA sequences as described above, a FASTA file containing the protein translation, a log file with alignments and detailed reports, a GFF file (General Feature Format) for use with genome software, an SVG file containing the graphical representation of the gene structure, and a YAML file which contains all information generated by WebScipio.

#### File upload

WebScipio can also be used as a viewer for Scipio result files. When a YAML file of a previous search is uploaded, all result views are available. This way, users can store the results of their searches locally and can look at them any time, instead of repeating extensive searches. WebScipio can thus also be used as a viewer for results obtained from Scipio, the command line version of the program.

### Web service

All functions of WebScipio can also be used remotely as a web service. This allows for seamless integration with existing programs. Many modern programming languages offer built-in support for the required protocols. This frees programmers from the need to locally install software and to download and store large genome files. By using this service, it is easy to augment existing data with information produced by WebScipio. In-house, we use WebScipio's web service to determine the gene structure of thousands of motor proteins stored in CyMoBase (, [24]). Storing the YAML data produced by WebScipio in a database and parsing it on demand is a powerful way of using this information. Ruby classes for conveniently handling the data structures can be obtained upon request.

### Cross-species analysis of myosins in Human, Orangutan, Common marmoset, and Mouse

The performance of Scipio and therefore WebScipio in an in-species scenario has been demonstrated already [18]. To test the capability of WebScipio when searching in species other than the origin of the query, we performed searches in four species, *Homo sapiens *(Human), as a reference, and *Pongo pygmaeus *(Orangutan), *Callithrix jacchus *(Common marmoset), and *Mus musculus *(Mouse) (ordered by increasing phylogenetic distance to Human). As queries we used a set of 40 manually annotated myosin protein sequences as described in ([25], see Additional file 1). For each species two searches were performed, one with the myosins from the species itself and one with the myosins from Human, giving a total of 280 searches.

**Table 1:** Overview of results.

**Target**	**Query from self**	**Query from Human**	**difference**
Human	99.97%/99.97%	n.a.	n.a.
Pongo	99.21%/99.02%	96.08%/95.46%	3.13%/3.56%
Callithrix	99.22%/99.11%	97.45%/96.38%	1.77%/2.73%
Mouse	98.21%/97.88%	93.36%/90.92%	4.85%/6.96%

Average matching percentages for 40 myosin protein sequences from Human. Percentages are (percentage of protein, not subtracting mismatches)/(percentage of amino acids found, subtracting mismatches). Searches were performed using a tile size of 5.

We are confident that the manually annotated sequences we used as queries contain the least possible number of errors, since we compared them to EST data and dozens of homologues sequences from other species. Thus, most discrepancies with their source genomes are due to sequencing errors and low coverage. For each search we provide two percentages: The first and most significant number is the percentage of protein stretches that could be mapped onto the genome, allowing for mismatches that naturally occur when doing cross-species searches. The second number is the percentage of individual amino acids that could be aligned with codons on the genome, counting all discrepancies.

**Figure 4:**
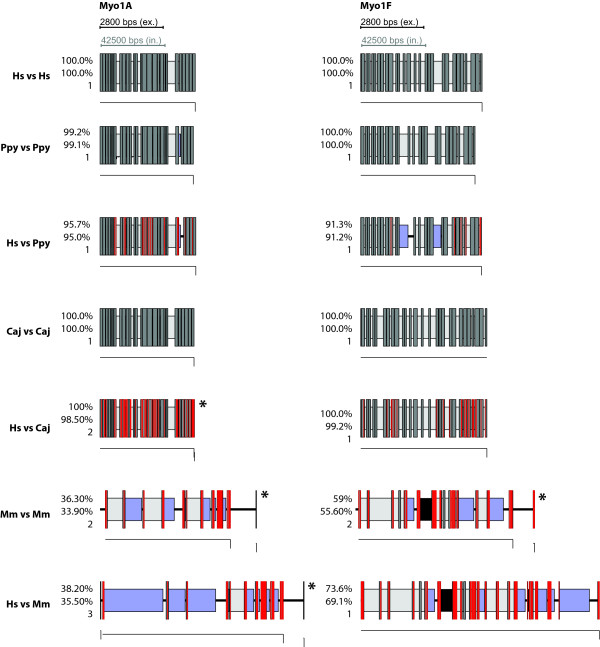
**Gene structures of Myo1A and Myo1F as determined by WebScipio**. Columns are the different variants of Myosin 1. Rows are either in-species or cross-species searches. Hs: *Homo sapiens*, Ppy: *Pongo pygmaeus*, Caj: *Callithrix jacchus*, Mm: *Mus musculus*. Numbers are: top: percentage of protein that could be mapped, middle: percentage of amino acids that could be mapped, buttom: number of contigs the predicted gene structure has been found on. Dark grey bars are introns, red bars are mismatches or frame shifts, light grey bars are introns with correctly determined splice sites, blue bars are introns without correctly determined splice sites, black bars are regions where amino acids could not be mapped onto the genome although there are nucleotides between the matching regions, central lines are amino acids that have no corresponding nucleotides. Thin lines beneath the gene structure depict the contigs on which the nucleotides have been found. For clarity, intron sequences have been scaled down by a factor of 15. Gene structures have been determined using a tile-size of 5. Gene structures with an asterisk (*) have been determined using a tile-size of 7.

**Figure 5:**
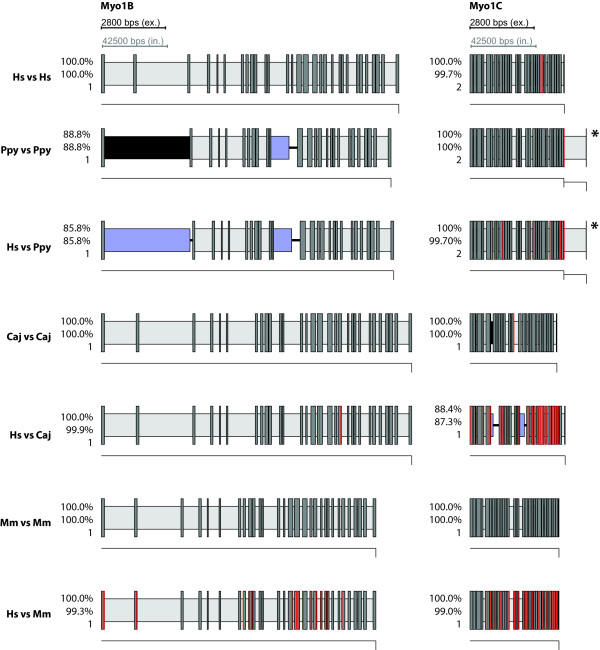
**Gene structures of Myo1B and Myo1C as determined by WebScipio**. The diagrams show the gene structures as in Figure 4.

**Figure 6:**
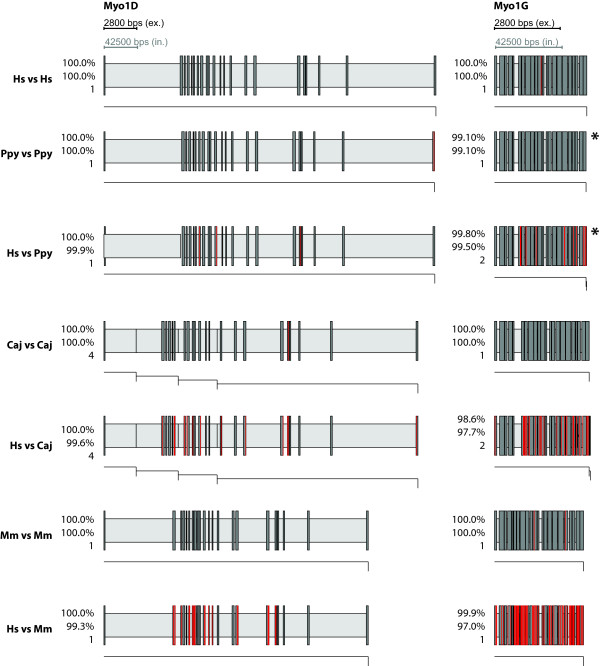
**Gene structures of Myo1D and Myo1G as determined by WebScipio**. The diagrams show the gene structures as in Figure 4. Note that the genes of Myo1D are depicted at half scale.

**Figure 7:**
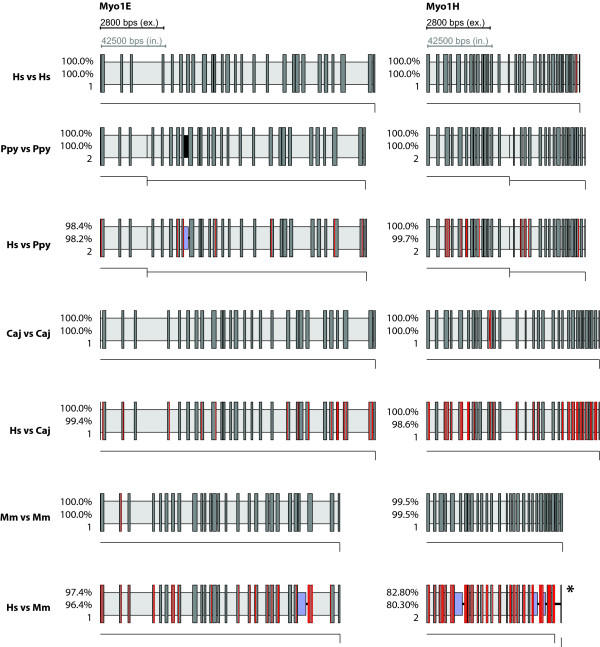
**Gene structures of Myo1E and Myo1H as determined by WebScipio**. The diagrams show the gene structures as in Figure 4.

As expected the agreement is very high when searching with queries from the target genome itself. But also when queries from Humans are used to search genomes from other species, WebScipio is able to map most of the genes correctly. For *Pongo *and *Callitrix*, on average, more than 96% percent of the Human query sequences were successfully found in the genomes. Even in Mouse, which is much more diverged, the difference between searching with a native query and searching with a query from Human is below 5%, meaning than in most cases, the structure of genes can be predicted with only minor gaps and inaccuracies. Figures 4, 5, 6, 7 show typical examples of in-species searches and cross-species searches for myosin class-I proteins. The searches against the source genome are all almost perfect matches. Only in the *Pongo *and mouse genomes, three genes could only be mapped with gaps (PpyMyo1B, MmMyo1A, MmMyo1F). Cross-species searches are, apart from the expected mismatches, almost as complete as the in-species searches.

For *Pongo*, three cross-species searches resulted in a reduction of the matching rate of less than five percent (Myo1A, Myo1B, Myo1E), three stayed the same (Myo1C, Myo1D, Myo1H), one got considerable worse (Myo1F), which can be attributed to the poor genome sequence in this region which contains stretches of N's. Using HsMyo1G as query, the *Pongo *homolog was found with better agreement since in this case WebScipio found a perfect 27 bp match on another contig, which was not present in the search results using the PpyMyo1G sequence as query.

In *Callitrix*, six out of the eight Human sequences where found with the same percentage as the *Callitrix *sequences (Myo1A, Myo1B, Myo1D, Myo1E, Myo1F, Myo1H) and two with minor losses (Myo1C, Myo1G). In the Mouse genome, three sequences where found with the same (Myo1B, Myo1C, Myo1D), two with very similar (Myo1E, Myo1G) agreement. For HsMyo1H, the percentage decreased considerable. Myo1F was not found; instead, it was matched with the gene of Myo1E, a close homolog. The reason for this probably is the high degree of fragmentation or the occurrence of large gaps in the region of the Myo1F gene. The observation that Human Myo1A can be slightly better mapped than the one from Mouse can be attributed to noise, since both hits have a low percentage of agreement (less than 40%).

### Future developments

For many applications it is useful to have information about the structures of genes in closely related species. Therefore, we plan on implementing a feature to select species based on a taxonomic tree. Also, we plan to include an option to search in several genomes simultaneously using the same query sequence.

## Conclusion

WebScipio is a service that maps protein queries onto a genome. All functionality and data resides on the server, so it is not required that the user installs software or downloads large files. WebScipio can be used through its web interface or as a web service, allowing for automated querying from within other software programs. The result of a search is a coherent prediction of the gene structure, consisting of a plausible combination of DNA stretches. Since WebScipio combines hits on different contigs, searches in genomes that are in an early stage of assembly are possible. The success rate of in-species searches is very high and the quality approaches the one of manual annotation. For cross-species searches, the tolerance of WebScipio makes it possible to find gene structures even in species with considerable phylogenetic distance to the source organism of the protein sequence.

We think that WebScipio can in many cases provide even non-specialists with gene structure predictions that are coherent and precise, therefore leading to more meaningful analyses.

## Availability and requirements

Project name: WebScipio

Project home page: 

Operating system: Platform independent

Programming language: Ruby

Software requirements: WebScipio has been tested with IE7, Firefox (≥2.0), and Safari.

License: WebScipio may be obtained upon request and used under a Creative Commons License.

Any restrictions to use by non-academics: Using WebScipio by non-academics requires permission.

## Authors' contributions

FO and MK set the requirements for the system. FO and HP wrote the software. FO and MK performed testing, and wrote the manuscript. OK improved the Scipio source code. SW supervised the implementation of Scipio. All authors read and approved the final version of the manuscript.

## Supplementary Material

Additional file 1 **Myosin sequences**. All myosin sequences used in the intra- and cross-species searches. Click here for file
